# Four-year antibiogram analysis of priority pathogens: guiding empirical therapy and monitoring resistance trends

**DOI:** 10.1128/spectrum.03457-25

**Published:** 2026-05-18

**Authors:** Krisha Panchal, Nasiruddin Shaikh, Tanmay Mehta, Jayshri Pethani

**Affiliations:** 1 Department of Microbiology, Smt. N.H.L Municipal Medical College 213456, Ahmedabad, Gujarat, India; Universita degli Studi dell'Insubria, Varese, Italy

**Keywords:** antibiogram, antimicrobial resistance, empirical therapy, bacterial pathogens, antimicrobial stewardship

## Abstract

Antibiograms are essential surveillance tools for monitoring institutional antimicrobial resistance (AMR) patterns and supporting antimicrobial stewardship programs (AMS). This study analyzed 4 years (2020–2023) of cumulative and subtraction antibiograms to describe local susceptibility trends among priority bacterial pathogens in a tertiary-care teaching hospital in western India. A retrospective observational study was conducted using the first-isolate-per-patient-per-year methodology in accordance with Clinical and Laboratory Standards Institute (CLSI) M39 guidelines. Antimicrobial susceptibility testing was performed using disk diffusion and automated minimum inhibitory concentration (MIC)-based systems, with colistin tested exclusively by broth microdilution. Cumulative and subtraction antibiograms were generated for *Escherichia coli*, *Klebsiella pneumoniae*, *Pseudomonas aeruginosa*, *Acinetobacter baumannii*, *Staphylococcus aureus*, and *Enterococcus* species. Data were analyzed descriptively. A total of 5,530 priority pathogen isolates were included. Gram-negative organisms demonstrated high resistance to fluoroquinolones, third-generation cephalosporins, and carbapenems, particularly *K. pneumoniae*, consistent with a substantial carbapenem-resistant Enterobacterales burden. Non-fermenters showed persistently low β-lactam susceptibility. Glycopeptides and linezolid retained high *in vitro* activity against gram-positive organisms. Colistin and tigecycline demonstrated high *in vitro* susceptibility across several multidrug-resistant organisms; however, year-to-year variability and fluctuating isolate numbers limit interpretation of temporal changes. This study highlights a sustained burden of AMR and underscores the importance of continuous, standardized antibiogram surveillance. Antibiogram data should inform stewardship strategies and institutional policy but must be interpreted alongside the clinical context and infection-control measures rather than used in isolation to determine empirical therapy.

**IMPORTANCE:**

Antimicrobial resistance (AMR) is a growing global health threat that makes common infections harder to treat and increases the risk of severe illness and death. Hospitals must continuously monitor local resistance patterns to ensure antibiotics remain effective. This study analyzes 4 years of hospital laboratory data to track how bacteria responded to commonly used antibiotics over time. The findings reveal a sustained burden of resistance, particularly among gram-negative organisms, highlighting the need for ongoing surveillance and responsible antibiotic use. By systematically evaluating cumulative and subtraction antibiograms, this work demonstrates how routine laboratory data can support antimicrobial stewardship programs (AMS) and guide institutional policy. Importantly, the study emphasizes that antibiograms should inform stewardship strategies rather than dictate treatment decisions in isolation. Continuous monitoring and careful interpretation of resistance trends are essential to preserving the effectiveness of existing antibiotics and strengthening infection-control efforts.

## INTRODUCTION

Antimicrobial resistance (AMR) represents a major and escalating threat to global public health, undermining the effective treatment of common bacterial infections and increasing morbidity, mortality, and healthcare costs. Hospitals, particularly tertiary-care centers, serve as focal points for the emergence and dissemination of resistant organisms due to high antimicrobial consumption, complex patient populations, and intensive medical interventions. Continuous local surveillance of antimicrobial susceptibility patterns is, therefore, essential to inform antimicrobial stewardship programs (AMS), infection-prevention strategies, and institutional antibiotic policies.

An antibiogram is a cumulative summary of antimicrobial susceptibility test (AST) results for selected pathogens over a defined time period within a healthcare facility. When constructed according to standardized methodologies, such as those outlined in the Clinical and Laboratory Standards Institute (CLSI) M39-A5 guideline, antibiograms provide a structured overview of local resistance epidemiology. Their primary function is descriptive: to highlight prevailing susceptibility patterns, detect emerging resistance trends, and support risk stratification in empirical prescribing decisions. Importantly, antibiograms are not designed to dictate empirical therapy in isolation as they do not account for clinical syndrome, infection severity, patient-specific risk factors, or ward-level epidemiology (1, 2).

Beyond routine cumulative antibiograms, subtraction antibiograms enable longitudinal assessment of changes in susceptibility by comparing consecutive time periods. This approach can help identify directional trends in resistance over time and may support evaluation of stewardship or infection-control interventions. However, interpretation of subtraction antibiograms requires caution as apparent changes may be influenced by fluctuations in isolate numbers, specimen mix, or healthcare utilization—factors that were particularly pronounced during the COVID-19 pandemic.

In low- and middle-income country (LMIC) settings, where access to molecular surveillance and real-time resistance mechanism data is often limited, well-constructed antibiograms remain a cornerstone of AMR monitoring. Nevertheless, their limitations—including lack of syndrome-specific stratification, absence of patient-level data, and potential sampling bias—must be explicitly acknowledged to avoid overinterpretation and unsafe clinical extrapolation.

The present study analyses 4 years (2020–2023) of cumulative and subtraction antibiogram data for priority gram-negative and gram-positive priority bacterial pathogens isolated in a tertiary-care teaching hospital in western India providing outdoor, indoor, and ICU services to a heterogeneous patient population. The objectives were to describe local antimicrobial susceptibility patterns, assess temporal changes in resistance profiles, and evaluate the potential utility of antibiogram data in supporting antimicrobial stewardship activities.

## MATERIALS AND METHODS

### Study design and setting

This retrospective observational study was conducted in the Department of Microbiology of a tertiary-care teaching hospital in western India. The hospital provides a wide range of clinical services, including medical and surgical inpatient care, intensive care units, and outpatient services, and caters to a heterogeneous patient population. Antimicrobial susceptibility data generated as part of routine clinical care between January 2020 and December 2023 were analyzed.

### Bacterial isolates and data collection

Clinical bacterial isolates were recovered from routine diagnostic specimens, including urine, blood, respiratory samples, pus, sterile body fluids, and wound swabs submitted to the microbiology laboratory. Identification and antimicrobial susceptibility testing (AST) results were extracted from the laboratory information system (LIS).

The priority pathogens analyzed were *Escherichia coli*, *Klebsiella pneumoniae*, *Pseudomonas aeruginosa*, *Acinetobacter baumannii*, *Staphylococcus aureus*, and *Enterococcus* species, selected based on their clinical relevance and burden of antimicrobial resistance. Isolate counts varied by year and organism, reflecting routine clinical sampling patterns and changes in healthcare utilization during the study period.

### Inclusion and exclusion criteria

Antimicrobial susceptibility data were included if at least 30 isolates per organism per year were available, in accordance with CLSI M39 recommendations for cumulative antibiogram construction (3). Only final, validated AST results were analyzed.

The following were excluded:

- Duplicate isolates from the same patient within the same calendar year
- Surveillance or environmental isolates
- Contaminants or non-clinically significant isolates
- AST results flagged as erroneous, advanced expert system errors or non-compliant with CLSI M100 interpretive criteria
- Organism-antimicrobial combinations with intrinsic resistance or without established clinical breakpoints or universally susceptible antibiotics showing resistance.

### Antimicrobial susceptibility testing

Bacterial identification and AST were performed as part of routine diagnostic workflows. Susceptibility testing was carried out using a combination of disk diffusion and VITEK compact-2 automated minimum inhibitory concentration (MIC)-based systems, depending on organism-drug combinations and laboratory protocols in place during the study period.

Colistin susceptibility testing was performed exclusively using broth microdilution (BMD).Disk diffusion and gradient diffusion methods were not used for colistin testing due to their known unreliability.

Interpretation of tigecycline AST reporting was done following the guidelines of the European Committee on Antimicrobial Susceptibility Testing (EUCAST) and United States Food and Drug Administration (USFDA) for all organisms except for Acinetobacter. For Acinetobacter tigecycline AST reporting, the MIC breakpoint based on the study by Pachón-Ibáñez ME et al. was used. Interpretation of AST for reporting for all other isolates was done following CLSI M100 standards applicable to the year of testing (4).

### Antibiogram construction

Cumulative antibiograms were generated annually using the guidelines using the first-isolate selection method (first-isolate per patient per species per year), as recommended by CLSI M39-A5, to minimize bias arising from repeat cultures. Susceptibility percentages were calculated based on the number of isolates tested for each organism-antimicrobial combination (3).

S: Susceptible indicates that the antimicrobial agent is clinically effective when used in standard therapeutic dose (3).

I: indicates that the isolate that is inhibited at the urinary site even when the antimicrobial agent is given at standard dosage as this antimicrobial agent is physiologically concentrated in urine (3)

Other abbreviations used are as follows : IR: intrinsically resistant; NBP: no breakpoint available; CIN: clinically ineffective; NT: not tested; NR: not routinely reported drug; NA: not applicable (<30 isolates) (3).

### Interpretation framework

Antibiogram data were interpreted using a color-coded system adapted from CLSI M39-A5 guidance:

- Green (>80% susceptible): high *in vitro* susceptibility—may (or may not) be the reasonable choice for empirical therapy, especially with high risk of mortality and morbidity (sepsis, meningitis, and ICU setting)
- Yellow (60%–80% susceptible): intermediate/moderate *in vitro* susceptibility—may (or may not) be the reasonable choice for empirical therapy in selected circumstances (OPD setting and stable patient)
- Red (<60% susceptible): low *in vitro* susceptibility—may not be the reasonable choice for empirical therapy

This color coding was intended to aid visual interpretation of resistance burden and support antimicrobial stewardship discussions. It was not used to define or recommend empirical therapy (3).

In accordance with journal accessibility requirements, color formatting was not used in tables. Instead, these categories were represented using alternative formatting:

- Gray shading was used to represent the “Green” category (>80%)
- Asterisk (*) was used to represent the “Yellow” category (60%–80%)
- Values without additional formatting correspond to the “Red” category (<60%)

Subtraction antibiograms combined OPD and IPD isolates as they are derived from routine cumulative facility-specific antibiograms. Subtraction antibiograms were constructed by calculating the numerical difference in susceptibility percentages between consecutive years for each organism-drug pair. These subtraction antibiograms were used solely to visualize directional changes over time and not to infer statistical significance (3).

Subtraction antibiograms compared S% changes across years, indicating susceptibility trends:

- Negative (**−**): Decreased susceptibility (rising resistance).
- Positive (**+**): Increased susceptibility (improved effectiveness) (3).
  For subtraction antibiograms, formatting conventions differed from cumulative antibiograms. Numerical changes in susceptibility between consecutive years were displayed as absolute percentage differences.
  To enhance readability without the use of color, gray shading in subtraction antibiograms was used exclusively to indicate positive changes (increase in susceptibility), while negative changes (decrease in susceptibility) were presented without shading and indicated by negative values.
  Thus, gray shading has different contextual meanings in cumulative and subtraction antibiograms, and interpretation should be made accordingly.

### Statistical analysis

Data were analyzed using descriptive statistics only, with susceptibility expressed as percentages. No inferential statistical analyses were performed. Consequently, observed year-to-year numerical differences should be interpreted cautiously, particularly in the context of variable isolate numbers, changing specimen distributions, and healthcare utilization fluctuations during the COVID-19 pandemic.

## RESULTS

### Overview of isolates and data completeness

Between January 2020 and December 2023, antimicrobial susceptibility data were analyzed for priority gram-negative and gram-positive priority bacterial pathogens isolated from routine clinical specimens. A total of 1,858 *Escherichia coli* and 1,573 *Klebsiella pneumoniae* isolates were included, representing the most frequently recovered gram-negative organisms. Among non-fermenters, *Pseudomonas aeruginosa* (*n* = 824) and *Acinetobacter baumannii* (*n* = 457) were analyzed. Gram-positive isolates included *Staphylococcus aureus* (*n* = 405) and *Enterococcus* species (*n* = 413).

Isolate numbers varied across study years and organisms, most notably for *A. baumannii*, which demonstrated a marked reduction in isolate counts following 2020. These fluctuations likely reflect changes in healthcare utilization, specimen submission patterns, and infection-control practices during the COVID-19 pandemic and subsequent periods. Consequently, year-to-year numerical differences should be interpreted with caution.

Annual cumulative antibiograms and subtraction antibiograms are presented in Tables 1 to 4.

**TABLE 1:** Gram-negative bacteria: % susceptibility of antibiotics as per routine cumulative antibiograms of years 2020–2023^*a*^

Bacteria	*E. coli*				*Klebsiella pneumoniae*				*Pseudomonas aeruginosa*				*Acinetobacter baumannii*			
Year	2020	2021	2022	2023	2020	2021	2022	2023	2020	2021	2022	2023	2020	2021	2022	2023
Total number of isolates	381	432	499	546	354	398	385	436	267	22	268	267	272	53	56	76
Ampicillin (S)	3	5.23	6.01	16	x	x	x	x	x	x	x	x	0	x	x	x
Ampicillin (S+I^)	3	5.5	6.48	16	x	x	x	x	x	x	x	x	x	x	x	x
Cefuroxime (S)	4.8	6.67	5.21	6.6	6.76	11.04	13.69	9.65	x	x	x	x	x	x	x	x
Cefuroxime (S+I^)	7	9	9	10	7.11	12.26	14.64	10.45	x	x	x	x	x	x	x	x
Cefoxitin (S)	22.3	42.85	55.56	48.15	5.45	27.27	44	38.89	x	x	x	x	0	x	x	x
Cefoxitin (S+I^)	26	49	65*	56	7.27	x	x	x	x	x	x	x	x	x	x	x
Cefixime (S)	5.6	12	25.45	20	3.7	14.3	26.9	25	x	x	x	x	x	x	x	0
Cefixime (S+I^)	6.2	12	27	22	3.7	x	x	x	x	x	x	x	x	x	x	x
Ceftriaxone (S)	9	12	13.18	17	9.01	13.26	18.92	15.01	x	x	x	x	2.7	x	0	0
Ceftriaxone (S+I^)	9.37	12	13	17	9.01	13.26	18.92	15.01	x	x	x	x	x	x	x	x
Ceftazidime (S)	20.47	37	45	46	5.56	11.27	20.29	13.56	46.18	44.8	38.81	59.92	3.13	3.33	0	3.03
Ceftazidime (S+I^)	20.5	37	45	46	5.56	14.08	26.09	18.64	48.85	44.8	39.95	59.92	3.54	2.78	1.96	3.03
Cefepime (S)	28.2	31.15	30.61	24.74	18	25	24.72	18.5	49.44	48.2	44.4	64.42*	6.3	5.88	0	2.67
Cefepime (S+SDD)	44	41	44	40	22.41	27.66	27.37	19.95	56.92	48.2	44.4	64.42*	6.6	5.88	0	2.67
Aztreonam (S)	x	34	35	38	13.34	14	18.18	7.5	x	x	41.17	26.31	x	0	x	x
Aztreonam (S+I^)	x	34	35	38	x	x	x	x	x	x	x	x	x	x	x	x
Amoxicillin Clav. Acid (S)	17.4	20.66	31.17	27.27	13.1	20.11	26.76	23.29	x	x	x	x	x	x	x	x
Amoxicillin Clav. Acid (S+I^)	27	29	38	38	17.26	24	31.47	27.6	x	x	x	x	x	x	x	x
Ticarcillin/clavulanic acid	x	47	56	x	x	x	x	x	18.01	21.82	23.62	x	5.61	2.78	0	x
Cefoperazone/sulbactam	44	41	50	57	21	26.13	31.39	29.16	47.19	44.59	38.58	61.66*	8.49	19.23	3.07	6.58
Piperacillin-tazobactam (S)	34	36.8	45.98	49.45	15.01	23.99	25.84	25	40.55	43.46	36.84	58.62	4.83	5.77	0	5.26
Piperacillin-tazobactam (S+SDD)	x	x	47	51	x	x	27.68	27.55	x	x	x	x	x	x	x	x
Meropenem (S)	65*	57.5	64.41*	73.9*	24.5	27.13	33.6	34.53	42.26	45.21	42.42	65.54*	4.81	5.77	1.79	2.63
Meropenem (S+I^)	66*	58	65*	75*	25.17	27.4	34.45	35.26	47.16	47.5	46.6	68.5*	x	7.69	3.6	6.58
Imipenem (S)	63.1*	54.8	64.18*	73.77*	23.67	26.33	33.61	31.81	46.99	45.95	44.78	65.41*	5.55	5.77	1.79	2.63
Imipenem (S+I^)	68.6*	60*	67*	75*	33.67	40.16	48.18	34.93	46.99	46.39	45.14	67.67*	x	5.77	3.6	2.63
Ertapenem (S)	53.9	52.78	59.69	65.25*	23.97	27.54	34.93	33.16	x	0	x	x	x	x	x	x
Ertapenem (S+I^)	56	54	62*	67*	23.97	28.11	35.52	33.92	x	x	x	x	x	x	x	x
Doripenem (S)	x	69*	72*	x	0	22	40.54	x	47.91	47.06	44.44	x	5.58	2.78	3.23	x
Doripenem (S+I^)	x	71*	72*	x	x	x	x	x	56.35	49.77	46.82	x	x	2.78	3.23	x
Gentamicin (S)	65.7*	65.19*	69.34*	76.37*	32.49	33	39.48	37.1	x	x	x	x	8.15	7.69	8.9	2.63
Gentamicin (S+I^)	66.8*	67*	70*	77.1*	38.98	36.5	42.33	37.55	x	x	x	x	10.37	11.54	8.93	6.58
Amikacin (S)	79*	83.1	85	88	36.44	42	45.45	39.76	52	53.6	46.27	65.1*	x	46.67	13.04	9.33
Amikacin (S+I^)	79.5*	83.76	85.37	90.89	39.8	44.7	74.28*	55.29	53.55	54	49.25	68.62*	x	46.67	30.4	10.67
Ciprofloxacin (S)	6.8	7.87	7.96	7.33	6.25	14.07	16.1	11.24	42.64	45.7	37.97	49.81	5.88	3.92	0	6.58
Ciprofloxacin (S+I^)	8.95	10.41	14	14	9.38	17.08	20.77	17.89	47.54	52.94	40.6	55.43	6.61	7.84	0	6.58
Levofloxacin (S)	9.34	2.94	5	4.25	9.69	10	6.82	4.88	36.36	43.44	33.96	45.63	7.07	2.86	1.96	6.15
Levofloxacin (S+I^)	5.34	19.11	21	22	9.69	16	29.54	7.31	39.01	46.15	39.17	51.33	9.6	14.28	11.76	9.23
Cotrimoxazole	36	33	38	43	27.4	28.89	40.26	37.39	x	x	x	x	14.39	13.46	7.14	10.67
Minocycline	90	54	77*	90	x	x	x	x	x	x	x	x	45.41	37.14	56	45.31
Tigecycline	98	98	98	100	37.88	37.53	44.76	59.47	x	x	x	x	81.92	60.78%*	70.59*	x
Colistin (I)	99.5	100	100	100	96	96.54	98.03	96.38	99.28	95.45	98.48	99.21	98.88	100	98.15	100

^*a*^ **Notes:***S+I^* indicates combined % of isolates that are **Susceptible (S)** and **Susceptible at increased exposure (I^)** at urinary sites. *I^* refers to “Susceptible, increased exposure”—indicating effective urinary tract concentrations even with standard dosing due to physiological drug accumulation in urine (as per CLSI M100). Only the **first isolate per patient per year** was included as per CLSI M39-A5 recommendations. Intrinsically resistant organism-drug combinations are excluded. Gray shaded cells correspond to the “Green” category (>80% susceptible). Values marked with * correspond to the “Yellow” category (60%–80% susceptible). Unmarked values correspond to the “Red” category (<60% susceptible). Categories are based on CLSI M39 guidance. Interpretation is descriptive and should not be used in isolation for empirical therapy decisions.

**TABLE 2:** Gram-positive bacteria: % susceptibility of antibiotics as per routine cumulative antibiograms of years 2020–2023^*a*^

Bacteria	*Staphylococcus aureus (all)*				*MRSA*				*MSSA*		*Enterococcus (all)*					*E. faecium*			*E. faecalis*	
Year	2020	2021	2022	2023	2020	2021	2022	2023	2022	2023	2020	2021	2022	2023	2020	2021	2022	2023	2021	2023
Number of isolates (n)	101	74	101	129	80	47	67	96	34	36	142	106	73	92	89	67	47	58	39	92
Oxacillin	22.77	36.49	34	26.36	x	x	x	x	x	x	x	x	x	x	x	x	x	x	x	x
Erythromycin	24.75	37.84	31.68	27.56	20	30	19	22	59	42	13	1	10	3	17	1	4	9	16	3
Clindamycin	44.55	56.76	55.45	53.91	36	49	40	46	82	80*	x	x	x	x	x	x	x	x	x	x
Tetracycline	78.22*	95.95	95.05	89.15	76*	98	93	86	100	97	20	9	11	15	27	12	11	19	5	15
Tigecycline	98.02	100	100	99.22	100	100	100	100	100	100	100	100	100	x	100	100	100	100	100	x
Gentamicin (S)	72.28*	80	63.35*	68.99*	x	63.83*	52	60*	91	97.2	x		x	x	x	x	x	x		x
Gentamicin (S+I^)	80.19	87.83	79.2*	73.64*	66*	72*	73.13*	65.62*	91	94	x	x	x	x	x	x	x	x	x	x
Ciprofloxacin (S)	3.96	5.4	3.96	6.2	x	0	0	3	15	19	3.52	4.71	3	x	x	1	2	x	10.25	x
Ciprofloxacin (S+I^)	3.96	4.05	4.95	7.75	4	2	1.49	2	12	14	4.2	6	3	x	2	1	2	x	13	x
Levofloxacin (S)	4.95	5.4	4.95	7.75	4	0	0	3	15	19	2.81	5.66	3	x	0	1	2	x	13.15	x
Levofloxacin (S+I^)	4.95	4.05	4.95	7.75	4	2	0	3	15	19	6	8	5	x	3	3	2	x	16	x
Cotrimoxazole	50	45.95	48.51	51.94	49	45	40	45	74*	69*	x	x	x	x	x	x	x	x	x	x
Vancomycin	99	97.3	99.01	100	99	98	99	100	100	100	82	66*	82	80	73*	54	74*	71*	87	80
Teicoplanin	99	98.65	100	98.45	99	98	100	98	100	100	76*	75*	85	79*	67*	63*	74*	67*	95	79*
Linezolid	99.01	100	99.01	99.22	99	100	99	99	100	100	90	75*	81	92	88	63*	70*	88	97	92
Daptomycin	83.67	90.54	92.86	90.4	83	91	89	87	100	100	65*	55	x	50	x	x	x	x	55	50
Rifampicin	84.16	95.89	95.92	96.09	83	94	91	95	100	100	x	x	x	x	x	x	x	x	x	x
																				

^*a*^ **Notes:**MRSA: methicillin-resistant *Staphylococcus aureus; MSSA: *methicillin-sensitive *Staphylococcus aureus; E. faecium: Enterococcus faecium; E. faecalis: Enterococcus faecalis.S+I^* indicates combined % of isolates that are **Susceptible (S)** and **Susceptible at increased exposure (I^)** at urinary sites. *I^* refers to “Susceptible, increased exposure”—indicating effective urinary tract concentrations even with standard dosing due to physiological drug accumulation in urine (as per CLSI M100). Only the **first isolate per patient per year** was included as per CLSI M39-A5 recommendations. Intrinsically resistant organism-drug combinations are excluded. Gray shaded cells correspond to the “Green” category (>80% susceptible). Values marked with * correspond to the “Yellow” category (60%–80% susceptible). Unmarked values correspond to the “Red” category (<60% susceptible). Categories are based on CLSI M39 guidance. Interpretation is descriptive and should not be used in isolation for empirical therapy decisions.

**TABLE 3:** Subtraction antibiograms for the priority pathogenic gram-negative bacteria^*a*^

Gram-negative bacteria	*E. coli*			*Klebsiella pneumoniae*			*Pseudomonas aeruginosa*			*Acinetobacter baumannii*		
Antibiotic	2021 to 2020	2022 to 2021	2023 to 2022	2021 to 2020	2022 to 2021	2023 to 2022	2021 to 2020	2022 to 2021	2023 to 2022	2021 to 2020	2022 to 2021	2023 to 2022
Ampicillin (S)	+2.23	+0.78	+9.99	-	-	-	-	-	-	-	-	-
Ampicillin (S+I^)	+2.5	+0.98	+9.52	-	-	-	-	-	-	-	-	-
Cefuroxime (S)	+1.87	−1.46	+1.39	+4.28	+2.65	−4.04	-	-	-	-	-	-
Cefuroxime (S+I^)	+2	0	+1	+5.15	+2.38	−4.19	-	-	-	-	-	-
Cefoxitin (S)	+20.55	+12.71	−7.41	+21.82	+16.73	−5.11	-	-	-	-	-	-
Cefoxitin (S+I^)	+23	+16	−9	-	-	-	-	-	-	-	-	-
Cefixime (S)	+6.4	+13.45	−5.45	+10.6	+12.6	−1.9	-	-	-	-	-	-
Cefixime (S+I^)	+5.8	+15	−5	-	-	-	-	-	-	-	-	-
Ceftriaxone (S)	+3	+1.18	+3.82	+4.25	+5.66	−3.91	-	-	-	-	-	0
Ceftriaxone (S+I^)	+2.63	+1	+4	+4.25	+5.66	−3.91	-	-	-	-	-	-
Ceftazidime (S)	+16.53	+8	+1	+5.71	+9.02	−6.73	−1.38	−5.99	+21.11	+0.2	−3.33	+3.03
Ceftazidime (S+I^)	+16.5	+8	+1	+8.52	+12.01	−7.45	−4.05	−4.85	+19.97	−0.76	−0.82	+1.07
Cefepime (S)	+2.95	−0.54	−5.87	+7	−0.28	−6.22	−1.24	−3.8	+20.02	−0.42	−5.88	+2.67
Cefepime (S+SDD)	-3	+3	−4	+5.25	−0.29	−7.42	−8.72	−3.8	+20.02	−0.72	−5.88	+2.67
Aztreonam (S)	-	+1	+3	+0.66	+4.18	−10.68	-	-	−14.86	-	-	-
Aztreonam (S+I^)	-	+1	+3	-	-	-	-	-	-	-	-	-
Amoxicillin Clav. Acid (S)	+3.26	+10.51	−3.9	+7.01	+6.65	−3.47	-	-	-	-	-	-
Amoxicillin Clav. Acid (S+I^)	+2	+9	0	+6.74	+7.47	−3.87	-	-	-	-	-	-
Ticarcillin/clavulanic acid	-	+9	-	-	-	-	+3.81	+1.8	-	−2.83	−2.78	-
Cefoperazone/sulbactam	-3	+9	+7	+5.13	+5.26	−2.23	−2.6	−6.01	+23.08	+10.74	−16.16	+3.51
Piperacillin-tazobactam (S)	+2.8	+9.18	+3.47	+8.98	+1.85	−0.84	+2.91	−6.62	+21.78	+0.94	−5.77	+5.26
Piperacillin-tazobactam (S+SDD)	-	-	+4	-	-	−0.13	-	-	-	-	-	-
Meropenem (S)	−7.5	+6.91	+9.49	+2.63	+6.47	+0.93	+2.95	−2.79	+23.12	+0.96	−3.98	+0.84
Meropenem (S+I^)	-8	+7	+10	+2.23	+7.05	+0.81	+0.34	−0.9	+21.9	-	−4.09	+2.98
Imipenem (S)	−8.3	+9.38	+9.59	+2.66	+7.28	−1.8	−1.04	−1.17	+20.63	+0.22	−3.98	+0.84
Imipenem (S+I^)	−8.6	+7	+8	+6.49	+8.02	−13.25	−0.6	−1.25	+22.53	-	−2.17	−0.97
Ertapenem (S)	−1.12	+6.91	+5.56	+3.57	+7.39	−1.77	-	-	-	-	-	-
Ertapenem (S+I^)	-2	+8	+5	+4.14	+7.41	−1.6	-	-	-	-	-	-
Doripenem (S)	-	+3	-	+22	+18.54	-	−0.85	−2.62	-	−2.8	+0.45	-
Doripenem (S+I^)	-	+1	-	-	-	-	−6.58	−2.95	-	-	+0.45	-
Gentamicin (S)	−0.51	+4.15	+7.03	+0.51	+6.48	−2.38	-	-	-	−0.46	+1.21	−6.27
Gentamicin (S+I^)	+0.2	+3	+7.1	−2.48	+5.83	−4.78	-	-	-	+1.17	−2.61	−2.35
Amikacin (S)	+4.1	+1.9	+3	+5.56	+3.45	−5.69	+1.6	−7.33	+18.83	-	−33.63	−3.71
Amikacin (S+I^)	+4.26	+1.61	+5.52	+4.9	+29.58	−18.99	+0.45	−4.75	+19.37	-	−16.27	−19.73
Ciprofloxacin (S)	+1.07	+0.09	−0.63	+7.82	+2.03	−4.86	+3.06	−7.73	+11.84	−1.96	−3.92	+6.58
Ciprofloxacin (S+I^)	+1.46	+3.59	0	+7.7	+3.69	−2.88	+5.4	−12.34	+14.83	+1.23	−7.84	+6.58
Levofloxacin (S)	−6.4	+2.06	−0.75	+0.31	−3.18	−1.94	+7.08	−9.48	+11.67	−4.21	−0.9	+4.19
Levofloxacin (S+I^)	+13.77	+1.89	+1	+6.31	+13.54	−22.23	+7.14	−6.98	+12.16	+4.68	−2.52	−2.53
Cotrimoxazole	−3	+5	+5	+1.49	+11.37	−2.87	-	-	-	−0.93	−6.32	+3.53
Minocycline	−36	+23	+13	-	-	-	-	-	-	−8.27	+18.86	−10.69
Tigecycline	0	0	+2	−0.35	+7.23	+14.71	-	-	-	−81.31	+69.98	-
Colistin (I)	+0.5	0	0	+0.54	+1.49	−1.65	−3.83	+3.03	+0.73	+1.12	−1.85	+1.85

^*a*^ **Notes:***S+I^* indicates combined % of isolates that are **Susceptible (S)** and **Susceptible at increased exposure (I^)** at urinary sites. *I^* refers to “Susceptible, increased exposure”—indicating effective urinary tract concentrations even with standard dosing due to physiological drug accumulation in urine (as per CLSI M100). Gray shading was used to highlight increases in susceptibility.

**TABLE 4:** Subtraction antibiograms for the priority pathogenic gram-positive bacteria^*a*^

Gram-positive bacteria	*Staphylococcus aureus*			MRSA			MSSA	*Enterococcus*			*E. faecium*		
Antibiotic	2021 to 2020	2022 to 2021	2023 to 2022	2021 to 2020	2022 to 2021	2023 to 2022	2023 to 2022	2021 to 2020	2022 to 2021	2023 to 2022	2021 to 2020	2022 to 2021	2023 to 2022
**Gentamicin (S)**	+7.72	−16.65	+5.64	-	−11.83	+8	+6.2	-	-	-	-	-	-
**Gentamicin (S+I^)**	+7.64	−8.63	−5.56	+6	+1.13	−7.51	+3	-	-	-	-	-	-
**Ciprofloxacin (S)**	+1.44	−1.44	+2.24	-	0	+3	+4	+1.19	−1.71	-	-	+1	-
**Ciprofloxacin (S+I^)**	+0.09	+0.9	+2.8	−2	−0.51	+0.51	+2	+1.8	−3	-	−1	+1	-
**Levofloxacin (S)**	+0.45	−0.45	+2.8	−4	0	+3	+4	+2.85	−2.66	-	+1	+1	-
**Levofloxacin (S+I^)**	−0.9	+0.9	+2.8	−2	−2	+3	+4	+2	−3	-	0	−1	-
Cotrimoxazole	−4.05	+2.56	+3.43	−4	−5	+5	−5	-	-	-	-	-	-
Tetracycline	+17.73	−0.9	−5.9	+22	−5	−7	−3	−11	+2	+4	−15	−1	+8
Tigecycline	+1.98	0	−0.78	0	0	0	0	0	0	-	0	0	0
Oxacillin	+13.72	−2.49	−7.64	-	-	-	-	-	-	-	-	-	-
Erythromycin	+13.09	−6.16	−4.12	+10	−11	+3	−17	−12	+9	−7	−16	+3	+5
Clindamycin	+12.21	−1.31	−1.54	+13	−9	+6	−2	-	-	-	-	-	-
Vancomycin	−1.7	+1.71	+0.99	−1	+1	+1	0	−16	+16	−2	−19	+20	−3
Teicoplanin	−0.35	+1.35	−1.55	−1	+2	−2	0	−1	+10	−6	−4	+11	−7
Linezolid	+0.99	−0.99	+0.21	+1	−1	0	0	−15	+6	+11	−25	+7	+18
Daptomycin	+6.87	+2.32	−2.46	+8	−2	−2	0	−10	-	-	-	-	-
Rifampicin	+11.73	+0.03	+0.17	+11	−3	+4	0	-	-	-	-	-	-

^*a*^ **Notes:**MRSA: methicillin-resistant *Staphylococcus aureus;MSSA:*methicillin-sensitive *Staphylococcus aureus; E. faecium: Enterococcus faecium; E. faecalis: Enterococcus faecalis.S+I^* indicates combined % of isolates that are **Susceptible (S)** and **Susceptible at increased exposure (I^)** at urinary sites. *I^* refers to “Susceptible, increased exposure”—indicating effective urinary tract concentrations even with standard dosing due to physiological drug accumulation in urine (as per CLSI M100). Gray shading was used to highlight increases in susceptibility.

### Escherichia coli

Across the study period, *E. coli* demonstrated persistently low susceptibility to fluoroquinolones and penicillin, cephalosporins and their beta-lactamase inhibitor combinations, as well as monobactams. Susceptibility to carbapenems remained moderate, with variation observed between years. Cotrimoxazole demonstrated low susceptibility with modest variation over time.

Aminoglycosides, particularly amikacin, retained relatively higher susceptibility compared with other antimicrobial classes, although year-to-year variability was observed.

High *in vitro* susceptibility to tigecycline and colistin was observed throughout the study period. These findings reflect laboratory susceptibility patterns only and do not account for clinical applicability or site-specific pharmacokinetic considerations (see Table 1).

### Klebsiella pneumoniae

*K. pneumoniae* exhibited consistently low susceptibility to carbapenems across all study years, with susceptibility rates remaining below 40%. This pattern is indicative of a substantial burden of carbapenem-resistant *K. pneumoniae* within the institution. Third-generation cephalosporins, aztreonam, fluoroquinolones, and cotrimoxazole, demonstrated low susceptibility throughout the study period.

Susceptibility to amikacin varied across years without a consistent directional trend. Tigecycline demonstrated low to moderate *in vitro* susceptibility with numerical variation between years. Colistin retained high *in vitro* susceptibility across all years analyzed (see Table 1).

### Pseudomonas aeruginosa

For *P. aeruginosa*, susceptibility to beta-lactams remained variable across the study period. Lower susceptibility persisted to carbapenem, cefepime, cefoperazone-sulbactam, and amikacin till 2022, but improved in 2023. Numerical increases in susceptibility to selected agents, including imipenem, meropenem, ceftazidime, and cefepime, were observed in later years; however, given the absence of inferential statistical testing and fluctuating isolate numbers, these differences should be interpreted descriptively rather than as definitive trends.

Colistin consistently showed high *in vitro* susceptibility across all years (see Table 1).

### Acinetobacter baumannii

*A. baumannii* demonstrated persistently low susceptibility to most beta-lactams, including carbapenems, beta-lactam/beta-lactamase inhibitor combinations, and minocycline. Susceptibility to tigecycline varied across years, with no consistent directional trend observed.

Colistin remained the antimicrobial agent with the highest *in vitro* susceptibility against *A. baumannii* throughout the study period. Interpretation of year-to-year differences is limited by small isolate numbers in certain years (see Table 1).

### Staphylococcus aureus

Among *S. aureus* isolates, high *in vitro* susceptibility to glycopeptides (vancomycin and teicoplanin), linezolid, daptomycin, and rifampicin was observed across all study years. Susceptibility to fluoroquinolones and macrolides remained low. Tetracycline showed a susceptibility of 76%–78% in 2020 to >95% in 2021 and 2022 and >85% in 2023. Gentamicin showed variable susceptibility over the years (60% to >80%).

Stratification by methicillin resistance status demonstrated expected differences in susceptibility profiles between MRSA and MSSA. However, MSSA-specific data were unavailable for 1 study year, limiting longitudinal interpretation for this subgroup (see Table 2).

### *Enterococcus* species

*Enterococcus* species demonstrated high *in vitro* susceptibility to linezolid, vancomycin, and tigecycline across the study period. For *Enterococcus faecium*, tigecycline retained consistently high *in vitro* activity, while linezolid susceptibility demonstrated numerical variability across years.

Vancomycin susceptibility showed variation over time among *Enterococcus* isolates. Interpretation of these findings is limited by the absence of clinical syndrome data and resistance mechanism analysis (see Table 2).

### Subtraction antibiograms

Subtraction antibiograms illustrated numerical changes in susceptibility percentages between consecutive years for several organism-antimicrobial combinations. While these visualizations highlight directional changes, they do not imply statistical significance and should be interpreted in the context of variable isolate numbers and changing specimen distributions (see Tables 3 and 4).

## DISCUSSION

This study provides a 4-year descriptive overview of antimicrobial susceptibility patterns among priority gram-negative and gram-positive bacterial pathogens isolated in a tertiary-care hospital. By analyzing routine cumulative and subtraction antibiograms, the study characterizes local resistance epidemiology and examines temporal variation in susceptibility within a stewardship-oriented framework. The findings demonstrate a substantial burden of antimicrobial resistance (AMR), particularly among gram-negative organisms, and reinforce the importance of continuous local surveillance and antimicrobial stewardship programs (AMS) (3, 5, 6).

### Gram-negative organisms and resistance epidemiology

The persistently low carbapenem susceptibility observed in *Klebsiella pneumoniae* throughout the study period is most consistent with endemic carbapenem-resistant Enterobacterales (CRE). Although minor numerical fluctuations were observed, carbapenem susceptibility remained well below thresholds that would suggest epidemiological recovery. In the Indian and global context, such patterns are commonly associated with dissemination of carbapenemase-producing strains, including NDM, OXA-48-like, and KPC enzymes (7–9). In the absence of molecular resistance testing, the present data remain epidemiologically descriptive; nevertheless, the consistently low susceptibility underscores the entrenched nature of CRE and its implications for clinical management and infection control. Low susceptibility to fluoroquinolones and cotrimoxazole, along with a declining trend, is consistent with prior reports highlighting fluoroquinolone resistance in *Klebsiella pneumoniae* due to mutations in quinolone resistance-determining regions (10).

*Escherichia coli* demonstrated sustained resistance to fluoroquinolones and third-generation cephalosporins, consistent with widespread extended-spectrum β-lactamase (ESBL) production reported across South Asia (11, 12). Moderate carbapenem susceptibility suggests partial preservation of activity; however, interpretation is limited by lack of syndrome-specific stratification. Aminoglycosides, particularly amikacin, showed steady upward susceptibility trends suggesting the need for monitoring aminoglycosides through AMS protocols despite being an Access group of antimicrobials as per AWaRe classification (6).

Among non-fermenting gram-negative bacilli, *Pseudomonas aeruginosa* exhibited variable susceptibility to antipseudomonal β-lactams and carbapenems. This variability is characteristic of *P. aeruginosa*, reflecting a combination of intrinsic and acquired resistance mechanisms, including efflux pump overexpression, porin loss, and inducible AmpC β-lactamase production (13, 14). Although numerical increases in susceptibility to selected agents were observed in later years, these changes cannot be causally attributed to stewardship or infection-control interventions in the absence of antimicrobial consumption or outcome data.

*Acinetobacter baumannii* demonstrated persistently low susceptibility to most β-lactams, including carbapenems, across all study years. This finding aligns with global reports describing multidrug-resistant *A. baumannii* driven by OXA-type carbapenemases, efflux mechanisms, and target-site alterations (15, 16). Interpretation of year-to-year variation is further limited by marked fluctuations in isolate numbers.

### Interpretation of reserve antimicrobials

High *in vitro* susceptibility to colistin and tigecycline was observed across all priority gram-negative organisms. These findings must be interpreted cautiously and strictly within a laboratory context. Colistin is considered a last-resort agent due to its nephrotoxicity and neurotoxicity, and the global emergence of plasmid-mediated resistance mechanisms, such as *mcr* genes, raises significant concerns regarding its sustainability (17, 18). Tigecycline, despite broad *in vitro* activity, has pharmacokinetic limitations, including low serum and urinary concentrations, which restrict its role in bloodstream and urinary tract infections (19). Consequently, high *in vitro* susceptibility of colistin and tigecycline does not equate to clinical suitability for empirical therapy without clinical context and antimicrobial stewardship, and inappropriate use of these agents risks accelerating resistance to critically important antimicrobials.

### Gram-positive organisms

Among gram-positive pathogens, *Staphylococcus aureus* (including MRSA) retained high *in vitro* susceptibility to glycopeptides, linezolid, daptomycin, rifampicin, and tigecycline consistent with their established roles in targeted therapy for resistant infections (20, 21). Persistent resistance to fluoroquinolones and macrolides, particularly among methicillin-resistant *S. aureus* (MRSA), reflects long-standing selective pressure associated with widespread antimicrobial use. Tetracycline susceptibility improved, reflecting decreased usage over the years and reducing the selection pressure.

*Enterococcus* species demonstrated high *in vitro* susceptibility to linezolid, vancomycin, and tigecycline. However, numerical variation in vancomycin susceptibility underscores the need for continued vigilance for vancomycin-resistant enterococci (VRE), which remain a significant nosocomial concern globally (22, 23). As with gram-negative organisms, these findings should not be extrapolated directly to empirical therapy decisions without a clinical correlation.

### National and global comparisons

The rising resistance to fluoroquinolones and beta-lactams mirrors patterns reported across India and globally (24). The consistent susceptibility of colistin and tigecycline is also in line with SENTRY and WHO data. Similar multicenter studies in India, such as those done by ICMR AMR surveillance, have shown comparable carbapenem resistance in *K. pneumoniae* and *A. baumannii*.

### Role and limitations of antibiogram-guided stewardship

Routine cumulative and subtraction antibiograms are valuable surveillance tools that summarize institutional susceptibility patterns, detect emergence of new resistance, and support as well as monitoring of AMS activities; however, their limitations are well recognized. They lack clinical granularity, do not account for infection syndrome or patient-level risk factors, and may be influenced by sampling bias and fluctuating isolate numbers (3, 5). Subtraction antibiograms can visualize directional changes over time but are particularly susceptible to misinterpretation when healthcare utilization or specimen changes, as observed during the COVID-19 pandemic.

Importantly, contemporary stewardship frameworks emphasize that antibiograms should inform principles rather than prescriptions. High resistance to commonly used agents should prompt reinforcement of antimicrobial-sparing strategies, early microbiological sampling, timely de-escalation, and robust infection-prevention measures, rather than escalation to restricted-line agents for empirical use (6, 25).

### Implications for antimicrobial stewardship and infection control

The findings of this study reinforce the necessity for integrated AMS and infection-control efforts in settings with a high prevalence of multidrug-resistant organisms. Persistent resistance underscores the need for robust contact precautions, including hand hygiene, isolation protocols, single-use patient-dedicated equipment, and personal protective equipment (PPE). Continuous local surveillance, combined with prescriber education, audit-and-feedback mechanisms, and alignment with national and international stewardship frameworks, is essential to preserve antimicrobial efficacy. An antibiogram can also guide antibiotic policy and pharmacy purchase policies in the institution. Antibiogram data contributed to various AMR surveillance networks as well as creating a national AMR database.

### Limitation and future recommendations

Our study conducted in a resource-limited setting is limited to a single center and lacks molecular resistance mechanism detection. Future studies incorporating syndrome-specific and enhanced antibiograms, antimicrobial consumption data, and molecular resistance profiling would further enhance the clinical relevance of antibiogram-based surveillance (5, 6).

### Conclusion

This 4-year analysis of routine cumulative and subtraction antibiograms provides a comprehensive overview of antimicrobial susceptibility patterns among priority bacterial pathogens in a tertiary-care hospital. The findings demonstrate a substantial and persistent burden of antimicrobial resistance, particularly among gram-negative organisms, underscoring the ongoing challenges faced by healthcare institutions in managing resistant infections.

The study highlights the value of continuous, standardized antibiogram surveillance as a core component of antimicrobial stewardship programs. When interpreted within their methodological limitations, antibiograms support institutional risk assessment, inform stewardship priorities, and contribute to infection-control planning. However, they should not be used in isolation to determine empirical therapy as clinical context, infection syndrome, patient-specific factors, and healthcare setting-level epidemiology are essential determinants of appropriate antimicrobial use.

Sustained surveillance, integration of antibiogram data into stewardship frameworks, and reinforcement of infection-prevention measures are critical to preserving the effectiveness of existing antimicrobials. Future work incorporating syndrome-specific analyses, antimicrobial consumption data, and molecular resistance profiling would further enhance the clinical applicability of antibiogram-based surveillance and strengthen institutional responses to antimicrobial resistance.
